# Comprehensive exploration of the genetic contribution of the dopaminergic and serotonergic pathways to psychiatric disorders

**DOI:** 10.1038/s41398-021-01771-3

**Published:** 2022-01-10

**Authors:** Judit Cabana-Domínguez, Bàrbara Torrico, Andreas Reif, Noèlia Fernàndez-Castillo, Bru Cormand

**Affiliations:** 1 Departament de Genètica, Microbiologia i Estadística, Facultat de Biologia, Universitat de Barcelona, Barcelona, Catalonia Spain; 2 Centro de Investigación Biomédica en Red de Enfermedades Raras (CIBERER), Instituto de Salud Carlos III, Barcelona, Spain; 3 Institut de Biomedicina de la Universitat de Barcelona (IBUB), Barcelona, Catalonia Spain; 4 Institut de Recerca Sant Joan de Déu (IR-SJD), Esplugues de Llobregat, Barcelona, Catalonia Spain; 5 Department of Psychiatry, Psychosomatic Medicine and Psychotherapy, University Hospital Frankfurt, Frankfurt am Main, Germany

**Keywords:** Psychiatric disorders, Genomics

## Abstract

Psychiatric disorders are highly prevalent and display considerable clinical and genetic overlap. Dopaminergic and serotonergic neurotransmission have been shown to play an important role in many psychiatric disorders. Here we aim to assess the genetic contribution of these systems to eight psychiatric disorders (attention-deficit hyperactivity disorder (ADHD), anorexia nervosa (ANO), autism spectrum disorder (ASD), bipolar disorder (BIP), major depression (MD), obsessive-compulsive disorder (OCD), schizophrenia (SCZ) and Tourette’s syndrome (TS)) using publicly available GWAS analyses performed by the Psychiatric Genomics Consortium that include more than 160,000 cases and 275,000 controls. To do so, we elaborated four different gene sets: two ‘wide’ selections for dopamine (DA) and for serotonin (SERT) using the Gene Ontology and KEGG pathways tools, and two’core’ selections for the same systems, manually curated. At the gene level, we found 67 genes from the DA and/or SERT gene sets significantly associated with one of the studied disorders, and 12 of them were associated with two different disorders. Gene-set analysis revealed significant associations for ADHD and ASD with the wide DA gene set, for BIP with the wide SERT gene set, and for MD with the core SERT set. Interestingly, interrogation of a cross-disorder GWAS meta-analysis of the eight psychiatric conditions displayed association with the wide DA gene set. To our knowledge, this is the first systematic examination of genes encoding proteins essential to the function of these two neurotransmitter systems in these disorders. Our results support a pleiotropic contribution of the dopaminergic and serotonergic systems in several psychiatric conditions.

## Introduction

Psychiatric disorders represent a major health problem, affecting 29.2% of the worldwide population at some point during their lifetime [1], and are associated with considerable distress and functional impairment [2]. They constitute a set of complex traits that result from the interaction of genetic and environmental risk factors, with heritability ranging from 40 to 80%, depending on the disorder, as estimated by twin studies [3].

Interestingly, psychiatric disorders display considerable clinical overlap among them [4–6], and the presence of comorbidity is associated with increased severity and difficulty of treatment. Recent studies have shown strong genetic correlations among psychiatric phenotypes [7, 8], and the latest genome-wide association study (GWAS) meta-analysis conducted on eight psychiatric disorders (attention-deficit hyperactivity disorder (ADHD), anorexia nervosa (ANO), autism spectrum disorder (ASD), bipolar disorder (BIP), major depression (MD), obsessive-compulsive disorder (OCD), schizophrenia (SCZ) and Tourette’s syndrome (TS)) found that 75% of the LD-independent associated regions (109 out of 146) were associated with more than one disorder [9]. These results suggest that the high levels of comorbidity found in psychiatric disorders may be explained, at least in part, by shared genetic risk factors, supporting the existence of a set of genes that confer relatively broad liability to psychiatric disorders [9].

Dopamine (DA) and serotonin (SERT) are two important neurotransmitters that participate in the regulation of a wide range of essential functions of the organism (e.g. motor control, cognition, motivation, regulation of emotions or reward), and have been related to the physiopathology and treatment of many psychiatric disorders.

Dopaminergic dysfunction has been described in ADHD, ASD, OCD, TS, SCZ, mood disorders, and substance use disorders (SUD) among others [10]. For example, the positive symptoms of SCZ seem to be associated with hyperdopaminergic neurotransmission, especially in the mesolimbic system, while the negative symptoms and cognitive deficits might be caused by hypodopaminergic activity in the mesocortical pathway [11]. Therefore, most of the antipsychotic treatments block the dopamine D_2_ receptor (e.g. chlorpromazine and haloperidol), and some of them are combined with serotonin 5-HT2A receptor antagonists (e.g. clozapine and risperidone). Moreover, several studies have reported a reduction of dopaminergic receptor density in several brain regions of ADHD patients [12], which is in agreement with the mechanism of action of the ADHD treatments, like methylphenidate or amphetamine, that enhance dopamine transmission in prefrontal cortex [13].

Regarding the serotonergic system, there is strong evidence supporting its role in MD and in other mood disorders based on available pharmacological treatments. The serotonin hypothesis of depression postulates that reduced serotonin signaling is a risk factor in its etiology [14, 15], which is supported by the most effective antidepressant treatment, based on the use of serotonin selective reuptake inhibitors (SSRIs), which inhibit the serotonin transporter increasing the extracellular levels of the neurotransmitter [16]. However, the causal mechanisms connecting low serotonin signaling and depression are still unknown. The role of the serotonergic system in other psychiatric disorders like anxiety, SCZ, ADHD, or ASD is still unclear and further studies are needed [17]. Even so, all these psychiatric conditions seem to be related to serotonin dysfunction, and many psychotropic drugs interfere more or less directly with this system [18].

The contribution of these neurotransmitter systems to several psychiatric conditions is well known, as shown by the fact that several standard pharmacological approaches target these systems. However, drugs used nowadays that target the monoaminergic system are very unspecific. Genetic studies might help to identify more specific drug targets that share efficacy but add specificity. Dopaminergic and serotonergic neurotransmission have been widely investigated through candidate-gene association studies in many psychiatric disorders, which have mainly assessed genetic variants in core genes encoding DA and SERT transporters, receptors, and metabolizers [19–22]. One of the most studied genes is *SLC6A3*, encoding the dopaminergic transporter (DAT), with multiple variants mapping at this locus, including rare variants, copy number variations (CNVs), variable number of tandem repeats (VNTRs), and single nucleotide polymorphisms (SNPs) that have been found associated with several psychiatric conditions [23]. Although genetic variants in the serotonin transporter (SERT), encoded by *SLC6A4*, have also been extensively studied in psychiatric behavioral genetics, these different studies failed to obtain consistent results [24]. Interestingly, the outcome of GWAS evidenced that the classical serotonergic and dopaminergic candidate genes, like transporters (*SLC6A3* and *SLC6A4*) and receptors are not significantly associated with any psychiatric disorder (https://www.ebi.ac.uk/gwas/home), with one exception, *DRD2*, that was found associated with both MD and SCZ [25–27].

In this study, we systematically explored the contribution of common variants in genes involved in dopaminergic and serotonergic neurotransmission in eight psychiatric disorders studied individually and in combination, using GWAS data from the Psychiatric Genomics Consortium (PGC).

## Materials and methods

### DA and SERT gene selection

**Fig. 1:**
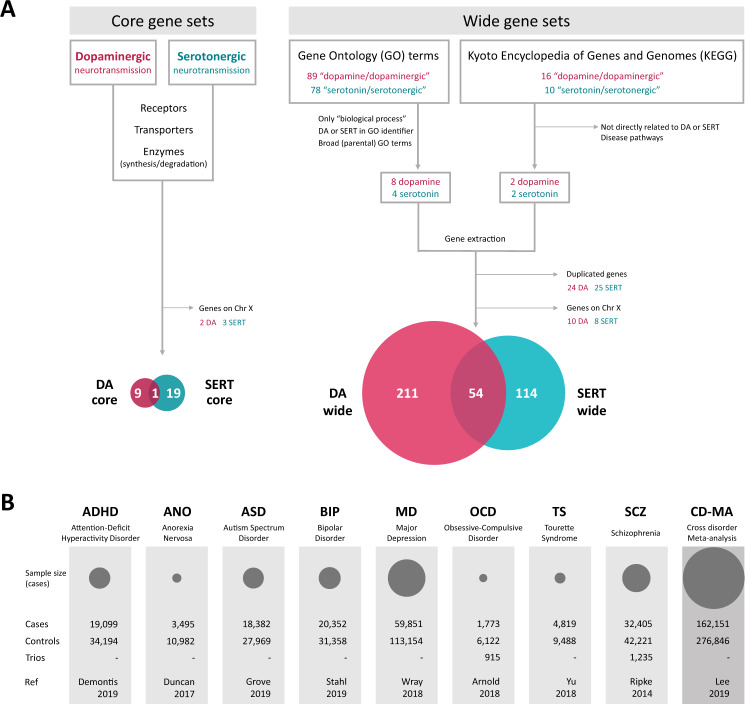
Workflow of the selection and analysis of dopamine and serotonin gene sets. **A** Strategy for the selection of the serotonin (SERT) and dopamine (DA) gene sets using Gene Ontology and KEGG’s pathways for the wide sets and manual curation for the core sets. All genes of the core gene sets are also included in their corresponding wide gene set. **B** Description of samples used in this study. The area of the circles is proportional to the sample size of the disorder.

We first obtained two core gene sets through manual curation (DA core with 12 genes, and SERT core with 23 genes), containing the well-known dopaminergic or serotonergic genes (neurotransmitter receptors: *DRD1-5*, *HTR1A-B*, *HTR1D-F*, *HTR2A-C*, *HTR3A-E*, *HTR4*, *HTR5A*, *HTR6*, *HTR7*; transporters: *DAT1/SLC6A3*, *5HTT/SLC6A4*; and enzymes involved in their anabolism or catabolism: *DDC*, *TH*, *TPH1*, *TPH2*, *DBH*, *COMT*, *MAOA*, *MAOB*) (Fig. 1A).

For a wide selection of genes encoding proteins involved in dopamine and serotonin neurotransmission and signaling pathways we used two main databases, GO and KEGG. We queried the GO database (Gene Ontology Consortium, http://www.geneontology.org/, September 2016) by the search terms “dopamine” or “serotonin/serotonergic”. The resulting list of GO terms (89 for dopamine and 78 for serotonin/serotonergic) was filtered to keep only those of “biological process” ontology source and with presence of the search term as part of the GO identifier. Then, we examined the hierarchical tree structure of the GO database to filter out the most specific terms (child terms) that were contained within broader ones (parental terms). The final GO terms selection included: “dopamine transport” (GO:0015872), “dopamine receptor signaling pathway” (GO:0007212), “dopamine receptor binding” (GO:0050780), “synaptic transmission, dopaminergic” (GO:0001963), “dopaminergic neuron axon guidance” (GO:0036514), “dopaminergic neuron differentiation” (GO:0071542), “response to dopamine” (GO:1903350), “dopamine metabolic process” (GO:0042417), “serotonin receptor signaling pathway” (GO:0007210), “serotonin transport” (GO:0006837), “serotonin metabolic process” (GO:0042428) and “serotonin production involved in inflammatory response” (GO:0002351). For the exploration of KEGG pathways (https://www.genome.jp/kegg/pathway.html, September 2016) we followed a similar procedure, using the terms “dopamine” or “serotonin” as keywords, and only those pathways more tightly related to neurotransmitters’ function were kept: “dopaminergic synapse” (hsa04728) and “tyrosine metabolism” (hsa00350), and “serotonergic synapse” (hsa04726) and “tryptophan metabolism” (hsa00380). The human entries obtained were then filtered for more inclusive and non-disease pathways.

We elaborated the dopaminergic (DA wide, 275 genes) and serotonergic (SERT wide, 176 genes) gene sets with human genes belonging to the final GO categories plus the selected KEGG pathways (Supplementary Table S1). Although GO and KEGG are based on experimentally supported annotations, we confirmed that these two lists contained the corresponding core genes. We also checked the expression of the included genes in the human brain using the GTEx database (https://gtexportal.org), and 95% of them were found to be expressed in brain tissues (Supplementary Table S2).

As the summary statistics used in our analyses did not include the X chromosome, we filtered out those genes from our gene sets. In the case of DA genes, 10 genes were removed: two core genes (*MAOA* and *MAOB*) and 8 other genes from the wide set (*ATP7A, AGTR2, FLNA, GPR50, GRIA3, HPRT1, PPP2R3B*, and *VEGFD)*. And 8 genes were excluded for SERT: three core genes (*MAOA, MAOB*, and *HTR2C*) and 5 other genes from the wide set (*ATP7A*, *ARAF, ASMT, CACNA1F*, and *GPM6B*) (Fig. 1A).

Finally, four gene lists were obtained: (i) DA core (10 genes); (ii) DA wide (265 genes); (iii) SERT core (20 genes); and (iv) SERT wide (168 genes) (Fig. 1A).

Gene symbols were converted to NCBI Entrez gene ID with the DAVID Gene ID Conversion Tool (https://david.ncifcrf.gov/conversion.jsp) for downstream analysis.

### Data used

We inspected the four DA and SERT gene sets in European samples of eight psychiatric disorders and in the meta-analysis of all of them (cross-disorder meta-analysis; CD-MA) [9] using publicly available GWAS summary statistics of attention-deficit hyperactivity disorder (ADHD) [28], anorexia nervosa (ANO) [29], autism spectrum disorder (ASD) [30], bipolar disorder (BIP) [31], major depression (MD) [32], obsessive-compulsive disorder (OCD) [33], schizophrenia (SCZ) [26] and Tourette’s syndrome (TS) [34] (Fig. 1B).

All of them were downloaded from the Psychiatric Genomics Consortium (PGC) webpage (https://www.med.unc.edu/pgc/results-and-downloads/). The MD and CD-MA summary statistics do not include 23andMe data used by the PGC in the MD GWAS.

### Gene-based and gene-set association analyses

Gene-based and gene-set association analyses of each phenotype were performed with MAGMA 1.06 [35] using the summary statistics from each individual GWAS meta-analysis and also from the cross-disorder GWAS, which is a meta-analysis of all eight conditions. For the gene-based analysis the SNP-wise mean model was used, in which the test statistic used was the sum of -log(SNP *p* value) for SNPs located within the transcribed region (defined on NCBI 37.3 gene definitions), with a 0 Kb window around genes. MAGMA accounts for gene size, number of SNPs in a gene and linkage disequilibrium between markers, using as a reference panel the European ancestry samples from the 1000 Genomes Project, phase 3 [36]. The resulting *p* values were corrected for multiple testing using False Discovery Rate (5% FDR).

Based on the gene-based *p* values we analysed the four sets of genes described above (DA wide, DA core, SERT wide, and SERT core). MAGMA applies a competitive test to analyse whether the genes of a gene set are more strongly associated with the trait than other genes, while correcting for a series of confounding effects such as gene length and size of the gene set. In our analyses, only genes on autosomes were included. Multiple testing corrections were performed for each disorder separately. As the Bonferroni correction is quite conservative for gene sets strongly overlapped, we used an empirical multiple testing correction implemented in MAGMA, based on a permutation procedure.

### Gene-based Manhattan plot

Results of the gene-based analysis of the cross-disorder meta-analysis were plotted in R using the package “qqman” [37].

### MetaXcan analyses

We considered all the SNPs located in each DA and SERT gene to infer whether the genetically determined expression of each of those genes correlates with the phenotypes considered in the study. These analyses were carried out on MetaXcan [38] using the summary statistics of each disorder. Prediction models were constructed considering SNPs located within ± 1 Mb from the transcription start site (TSS) of each gene and were trained with RNA-Seq data of 13 GTEx (release V7) brain tissues and whole blood [39]. The SNP covariance matrices were generated using the 1000 Genomes Project Phase 3 [36] EUR genotypes of the prediction model SNPs. For each brain tissue, the threshold for significance was calculated using the Bonferroni correction for multiple testing.

## Results

Dopaminergic and serotonergic neurotransmission have been pointed out as involved in many psychiatric disorders. In the present study, we aimed to assess the genetic contribution of these two monoaminergic systems to eight different psychiatric disorders (ADHD, ANO, ASD, BIP, MD, OCD, SCZ, and TS) as well as to the meta-analysis of all of them (CD-MA). For that purpose, we elaborated four different gene sets (DA core, 10 genes; DA wide, 265 genes; SERT core, 20 genes; and SERT wide, 168 genes; Fig. 1A and Supplementary Table S1) that were subsequently interrogated in the summary statistics from nine case-control GWAS meta-analyses (Fig. 1B). Importantly, we found some overlap between the wide datasets, with about 20% of genes in the DA gene sets present also in SERT, and 32% the other way round. One gene from the wide DA gene set (*KIF5C*) and two from the wide SERT gene set (*OR11H7* and *OR10J6P*) were not present in any of the GWAS datasets inspected.

### DA and SERT genes associated with psychiatric disorders

**Fig. 2:**
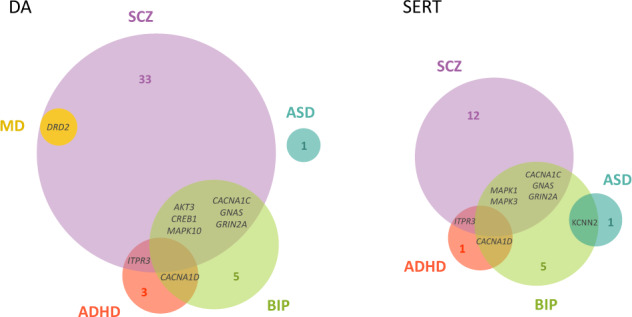
Dopaminergic and serotonergic genes associated with the studied disorders. Venn diagrams of the significantly associated DA and SERT genes across the studied disorders (5% False Discovery Rate). Gene names are only provided for those genes significantly associated with more than one disorder (overlapping genes). All the genes overlapping across phenotypes are significantly associated with the phenotype in the cross-disorder meta-analysis, except for *GNAS*. The area of circles is proportional to the number of significantly associated genes in each disorder. DA dopaminergic gene set, SERT serotonergic gene set, ADHD Attention-Deficit/Hyperactivity Disorder, ASD Autism Spectrum Disorder, BIP Bipolar Disorder, MD Major Depression, SCZ Schizophrenia.

**Fig. 3:**
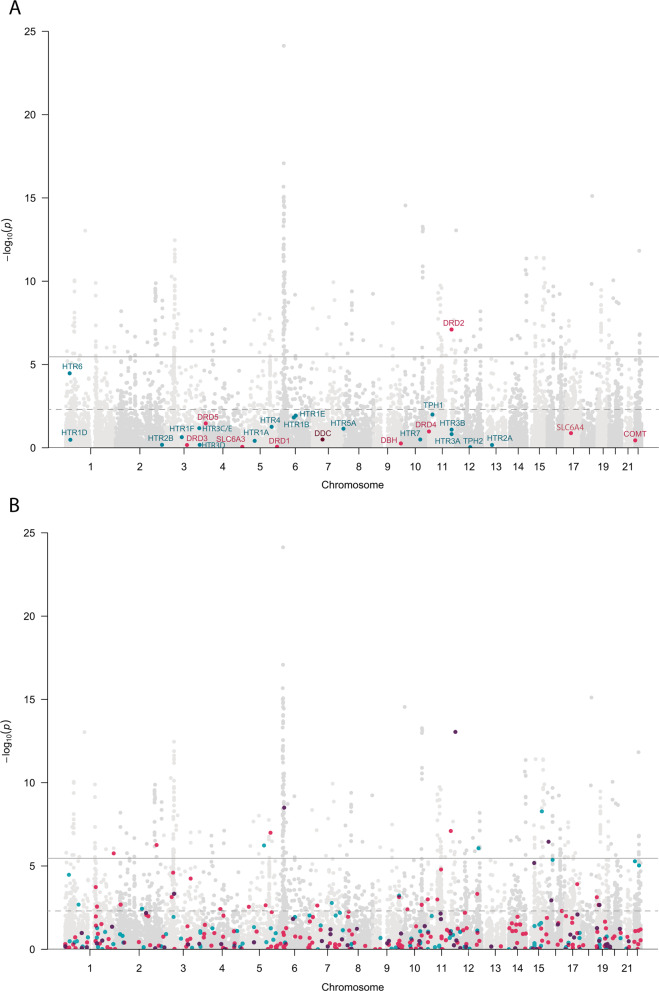
Manhattan plots of the gene-based association analysis performed on the cross-disorder GWAS meta-analysis data. Represented (**A**) DA core and SERT core genes, and (**B**) DA wide and SERT wide gene sets. Colored dots correspond to DA genes in pink, SERT genes in blue and overlapping genes in purple. Continuous line: threshold for Bonferroni significance (*P* = 2.8E-06). Discontinuous line: threshold for 5% False Discovery Rate (*P* = 4.9E-03).

At the gene-wide level, we found 67 genes from DA and/or SERT gene sets significantly associated (5% FDR) with at least one of the studied disorders, and 309 genes were not associated with any of them (Supplementary Table S3). Interestingly, twelve of these genes were associated with two different disorders, eight of them with SCZ and BIP (Fig. 2 and Supplementary Table S3). Eleven out of these twelve genes also showed a significant association with the CD-MA phenotype, *DRD2* from the DA core gene set among them. Interestingly, five out of these twelve genes (*CACNA1C, CACNA1D, GNAS, GRIN2A* and *ITR3*) belong to both the DA and SERT gene sets, highlighting the importance of those genes that are involved in both monoamine pathways (Supplementary Table S3). Then, we plotted the gene-based results of the cross-disorder meta-analysis in a Manhattan plot and highlighted the DA core and SERT core genes, to visualize the performance of these genes in the combination of all the psychiatric conditions considered in the study (Fig. 3A). As shown in the figure, only the *DRD2* gene overcomes the Bonferroni correction for multiple testing, and *HTR6* surpasses 5% FDR. Then, we repeated the analysis by highlighting the DA and SERT wide genes and found 10 genes with associations that overcome the Bonferroni correction and 32 additional genes overcoming 5% FDR (Fig. 3B) and Supplementary Table S3). Among them, *CACNA1C*, present in both DA and SERT gene lists, was the one showing the strongest association in the cross-disorder meta-analysis (raw *P* = 8.9E-14, *P*adj = 7.8E-11).

### Gene expression correlates in brain regions in psychiatric disorders

In addition, to identify genes which expression correlates with the phenotypes included in the study, we performed MetaXcan analysis, considering only those phenotypes that show significant association with DA or SERT genes (ADHD, ASD, MD, SCZ, and CD-MA). Taking into account the genes associated at gene level with a given disorder, we found that the expression of *CTNNB1* significantly correlates with ADHD in the amygdala (*P* = 8.9E-04, Z = −3.3246), *ATF6B* with SCZ in the spinal cord (*P* = 1.6E-06, Z = 4.7925) and *DNM1* and *ITPR3* with the phenotype in the CD-MA in the cortex and caudate, respectively (*P* = 2.6E-04, Z = 3.6516 and *P* = 1.8E-05, Z = −4.2860) (Table 1 and Supplementary Tables S4–S16). Interestingly, the expression of the *DNM1* gene in the cortex also correlates with ADHD (*P* = 8.5E-03, Z = 2.6308), ASD (*P* = 5.9E-03, Z = 2.7524), MD (*P* = 3.0E-03, Z = 2.9630) and SCZ (*P* = 0.0312, *Z* = 2.1549) in the same direction, and this gene was found nominally associated with all these disorders in the gene-based analysis (raw *P*_ADHD_ = 2.5E-04, raw *P*_ASD_ = 3.6E-03, raw *P*_MD_ = 1.5E-03 and raw *P*_SCZ_ = 0.0156). It is important to note that for most DA and SERT genes (ranging from 77% to 93.6% depending on the tissue and disorder) we could not retrieve information from MetaXcan because it only provides data about those gene models where the predictive power is good enough. For that reason, we decided to perform the analysis also on whole blood as the expression models are better for this tissue and we could retrieve information for about 30% of DA/SERT genes. This analysis highlighted additional genes (*ARNTL, FZD3*, *PPP1CB, PPP2R3C* and *PRMT5*) for SCZ and BIP, and supported some of the results obtained in brain tissues (Table 1 and Supplementary Table S17).

**Table 1:** MetaXcan prediction of gene expression effects on the studied disorders for brain tissues and whole blood.

Gene symbol	entrezID	Gene sets		Brain tissue^a^	Disorder	Z-score	*p* value	N SNPs in model	N SNPs used	Predicted R^2^
*ATF6B*	1388	DA		Spinal cord	SCZ	4.7924	**1.6E-06**	11	5	0.1466
				Whole blood	SCZ	−4.6153	**3.9E-06**	15	5	0.0246
*ARNTL*	406	DA		Whole blood	BIP	−3.0427	2.3E-03	16	16	0.1567
*CACNA1C*	775	DA	SERT	Cerebellum	CD-MA	−3.0166	2.6E-03	51	41	0.2435
					SCZ	−2.4867	0.0129	51	50	0.2435
*CACNA1D*	776	DA	SERT	Cerebellar hemisphere	ADHD	−2.2252	0.0261	107	89	0.0988
*CTNNB1*	1499	DA		Amygdala	ADHD	−3.3246	**8.9E-04**	36	35	0.1692
				Caudate	ADHD	−2.8011	5.1E-03	12	12	0.0789
				Putamen	ADHD	−3.0637	2.2E-03	28	27	0.1740
				Spinal cord	ADHD	−2.2299	0.0258	47	32	0.1377
				Whole blood	ADHD	3.9041	**9.5E-05**	5	5	0.0392
*CYP2D6*	1565		SERT	Amygdala	CD-MA	2.5247	0.1158	15	15	0.2419
					SCZ	2.3181	0.0204	15	15	0.2419
				Whole blood	SCZ	−5.1188	**3.1E-07**	23	23	0.4488
*DNM1*	1759	DA		Cortex	ADHD	2.6308	8.5E-03	3	3	0.0502
					ASD	2.7524	5.9E-03	3	3	0.0502
					CD-MA	3.6516	**2.6E-04**	3	3	0.0502
					MD	2.9630	3.0E-03	3	3	0.0502
					SCZ	2.1548	0.0312	3	3	0.0502
*FLOT1*	10211	DA		Cerebellar hemisphere	SCZ	−2.2126	0.0269	79	5	0.1378
*FZD3*	7976	DA		Whole blood	SCZ	−3.0575	2.2E-03	14	14	0.0873
*GCH1*	2643	DA		Cerebellum	SCZ	−1.1404	0.0490	103	102	0.0552
*GNB2*	2783		SERT	Spinal cord	CD-MA	−2.8822	3.9E-03	31	29	0.0579
*GRIN2A*	2903	DA	SERT	Spinal cord	BIP	−2.6559	7.9E-03	3	3	0.2636
*HTR6*	3362		SERT^b^	Anterior cingulate	BIP	2.4006	0.0164	58	58	0.0968
					CD-MA	2.4828	0.1303	58	49	0.0968
				Whole blood	BIP	−3.1707	1.5E-03	31	30	0.2042
*ITPR3*	3710	DA	SERT	Caudate	CD-MA	−4.2860	**1.8E-05**	4	4	0.0867
				Whole blood	ADHD	2.3412	0.0192	29	28	0.2384
*MAPK3*	5595		SERT	Hippocampus	SCZ	2.0050	0.0449	17	17	0.0734
				Whole blood	BIP	2.8559	4.3E-03	12	12	0.2902
					SCZ	5.4086	**6.3E-08**	12	11	0.2902
*MAPK10*	5602	DA		Anterior cingulate	BIP	2.3373	0.0194	15	15	0.1481
					CD-MA	2.8561	0.0344	15	15	0.1481
				Caudate	BIP	2.0394	0.0414	39	39	0.1546
					CD-MA	2.1152	0.0344	39	32	0.1546
				Cortex	BIP	2.6829	7.3E-03	28	28	0.0880
					CD-MA	2.6987	7.0E-03	28	22	0.0880
				Nucleus accumbens	BIP	2.1524	0.0314	35	35	0.1187
					CD-MA	2.3767	0.0175	35	33	0.1187
*PPP1CB*	5500	DA		Whole blood	SCZ	3.7165	**2.0E-04**	23	23	0.1395
*PPP2R3C*	55012	DA		Whole blood	SCZ	4.9201	**8.6E-07**	20	19	0.4025
*PRMT5*	10419	DA		Whole blood	SCZ	−2.8138	4.9E-03	23	22	0.2572

In bold: Significant *p* values overcoming the Bonferroni correction for multiple testing.

*ADHD* Attention-deficit/hyperactivity disorder, *ASD* Autism spectrum disorder, *BIP* Bipolar disorderm, *MD* Major depression, *SCZ* Schizophrenia, *CD-MA* Cross-disorder meta-analysis.

^a^Prediction models were only available for some tissues and genes; Z-score: Number of standard deviations change in gene expression; *p* value: Significance of the association between predicted expression levels and the disorder; N SNPs in model: Number of SNPs used in the training of prediction models for each gene; N SNPs used: Number of SNPs used from the corresponding GWAS summary statistics; Predicted R2: Correlation between the predicted and observed gene expression during prediction model training; DA: Wide dopaminergic gene set; SERT: Wide serotonergic gene set.

^b^Gene present in SERT core gene set.

### DA and SERT gene sets associated with psychiatric disorders

**Fig. 4:**
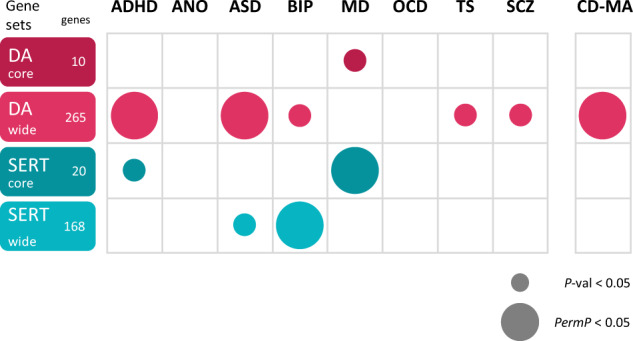
Results of the MAGMA gene-set association analysis on every phenotype under study. A small circle represents nominally significant *p* value, a big circle represents significant *p* value after the empirical multiple testing correction implemented in MAGMA. DA dopaminergic gene set, SERT serotonergic gene set, ADHD Attention-deficit/hyperactivity disorder, ANO Anorexia nervosa, ASD Autism spectrum disorder, BIP Bipolar disorder, MD Major depression, OCD Obsessive-compulsive disorder, TS Tourette’s syndrome SCZ Schizophrenia, CD-MA Cross- disorder.

Finally, we performed a gene-set analysis that revealed interesting significant associations. The DA wide gene set was significantly associated with ADHD (*P*_perm_ = 0.023) and ASD (*P*_perm_ = 0.025), and the SERT wide gene set with BIP (*P*_perm_ = 0.015). Besides, the SERT core (*P*_perm_ = 0.018) gene set was significantly associated with MD (Fig. 4 and Supplementary Table S18). Interrogation of the cross-disorder GWAS meta-analysis displayed association with the DA wide gene set (*P*_perm_ = 0.044). It should be mentioned that the DA wide gene set was nominally associated also with BIP, TS, and SCZ, in line with the results obtained in the cross-disorder analysis for this neurotransmitter system (Fig. 4). No significant results were found for ANO, OCD, and TS in either the gene-based or in the gene-set analyses after multiple testing correction, maybe due to limited sample sizes (number of patients below 5000; Fig. 1B).

## Discussion

Dopaminergic (DA) and serotonergic (SERT) functions represent good candidates for psychiatric disorders, mainly on the basis of the effectiveness of several pharmacological treatments and their target molecules [10, 23, 40–42]. Common genetic variation of genes encoding key participants of these two neurotransmission systems (receptors, transporters, and enzymes) has been broadly studied in psychiatric disorders, mainly through candidate-gene association studies, with several positive findings [23, 43–45]. However, except for a few cases [25–27], these hits have not been replicated by GWAS, which raises questions on how alterations in these two neurotransmitter systems can actually be causally related to psychiatric conditions, but also on the value of genome-wide significance for functional variation [46].

To our knowledge, this is the first systematic study of the DA and SERT neurotransmitter systems in eight psychiatric disorders (ADHD, ANO, ASD, BIP, MD, OCD, SCZ, and TS) using data from GWAS meta-analyses that include large number of samples. Here, we assess the contribution of genetic variation not only of genes encoding the core participants of DA and SERT functions, but also of a comprehensive list of genes involved in DA and SERT neurotransmission obtained by a systematic database search.

Our results show a contribution to psychiatric disorders of both dopaminergic and serotonergic systems at genetic level. We found several DA and SERT genes associated with some psychiatric disorders, twelve of them with two conditions, and also in the cross-disorder meta-analysis, which underscores the relevance of genetic risk factors in these genes for psychiatric disorders. Among the associated genes we identified the DA core gene coding for the dopamine receptor D2, *DRD2*, which has been widely studied as a candidate for different psychiatric disorders with some positive findings [47–51], especially in SCZ (recently meta-analyzed by [52]). The association between *DRD2* and SCZ observed through candidate-gene studies has subsequently been confirmed through GWAS [26]. Interestingly, a recent GWAS identified association of polymorphisms in *DRD2* with depressive symptoms, neuroticism and different traits of the well-being spectrum [53, 54]. These findings are in agreement with ours, obtaining associations of *DRD2* with SCZ and MD (Fig. 2 and Supplementary Table S3). Another interesting gene that encodes the calcium voltage-gated channel subunit alpha1 C (*CACNA1C*), present in both the DA and SERT wide gene lists and found associated by us with BIP and with SCZ (disorders with a very high genetic correlation, rg = 0.7 [9]), displayed the strongest association in the cross-disorder meta-analysis. Noteworthy, *CACNA1C* polymorphisms have been consistently associated with BIP and SCZ, among other psychiatric disorders [55, 56]. Mutations in this gene have been related to Timothy Syndrome, a monogenic disorder that affects multiple organs and presents with cognitive impairment, major developmental delays, and autism-like behaviors [57]. We also identified the potassium calcium-activated channel subfamily N member 2 (*KCNN2*) gene, from the SERT wide gene set, associated with BIP and with ASD, as previously described in two studies [30, 31].

Interestingly, two genes emerged from the analysis of gene expression using MetaXcan. First, the gene for inositol 1,4,5-trisphosphate receptor type 3 (*ITPR3*), found associated with ADHD, SCZ and the CD-MA at gene level in our study, which expression would be altered in the caudate of patients from the CD-MA and in whole blood from ADHD cases. The caudate nucleus is a crucial component of the ventral striatum, part of the basal ganglia that control motor, cognitive control, motivational and emotional processing [58], functions involved in the physiopathology of many psychiatric conditions. Several studies have found alterations both in the volume of the caudate nucleus and in its functional connections with other brain regions in many psychiatric disorders [59–61]. Interestingly, the *ITPR3* gene has been associated to type 1 diabetes [62], which curiously shows a correlation with risk of ADHD in the offspring [63–65]. The other gene that was pointed by this analysis was dynamin 1 (*DNM1*), which expression significantly correlates not only with the CD-MA, but also with ADHD, ASD, MD, and SCZ, in all cases in the cortex and in the same direction. Rare variants in *DNM1* have been identified in patients with a mendelian phenotype, epileptic encephalopathy [66], and in some cases of intellectual disability with seizures [67].

In the cross-disorder meta-analysis study [9], the eight psychiatric disorders used were classified into three groups using exploratory factor analysis (EFA): disorders characterized by compulsive/perfectionistic behaviors (AN, OCD, and TS), mood and psychotic disorders (MD, BIP, and SCZ), and early-onset neurodevelopmental disorders (ASD, ADHD, TS, as well as MD). Our results are in line with this classification, as we identified association of the serotonergic gene sets with BIP and MD, included in the same group, whereas the dopaminergic set was associated with ADHD and ASD, two disorders that conform the third factor.

Although SERT dysfunction has been proposed to be the common denominator in a wide range of neuropsychiatric illnesses [66], our gene-set results link this neurotransmitter system only with BIP (wide gene set) and with MD (core gene set). SERT signaling has been associated with concrete alterations in the intrinsic activity of the brain: an increase of the default-mode network and a decrease of the sensorimotor network. Interestingly, MD is characterized by an increase of internally focused thoughts and an inhibited psychomotor behavior and affectivity, an imbalance that has been also detected in BIP [66], but not in SCZ, the third of this genetically-correlated group of psychiatric disorders [9].

Gene-set analysis of the DA system showed association of the wide selection with ADHD and with ASD, two disorders that co-occur frequently [68] and with a high genetic correlation (rg = 0.37) [9]. Association of dopaminergic genes with these psychiatric conditions, especially the DA core genes, has been broadly studied using different strategies, including candidate-gene association studies or exome/genome sequencing [69–72]. Interestingly, the cross-disorder meta-analysis displayed association with the DA wide gene set, underscoring the importance that this neurotransmitter has in many psychiatric disorders [73, 74]. Finally, it is important to note that for SCZ, none of the studied gene sets were found significantly associated with the disorder, despite the large sample size of this study and the proven role of these neurotransmitter systems from pharmacological studies. However, we found many DA/SERT genes associated with SCZ at gene level (54 out of 376) and a nominal association with the DA wide gene set, highlighting the importance of this pathway in the pathophysiology of this disorder. Further studies are needed to confirm these results.

The primary drug target for antidepressants, antipsychotics, and stimulants are proteins that have a role in serotonin and dopamine neurotransmission and, accordingly, are encoded by genes belonging to these gene sets in our analysis. While serotonergic drugs have traditionally been used in affective disorders (e.g., selective serotonin reuptake inhibitors targeting the serotonin transporter) and dopaminergic drugs are used in schizophrenia (dopamine receptor antagonists) and ADHD (targeting the dopamine transporter), such distinction becomes more and more blurred. Compounds such as quetiapine or aripiprazole also affect monoaminergic transmitter systems, but are prescribed in depression, bipolar disorder, and schizophrenia. Therefore, the traditional grouping became obsolete and it was replaced by the Neuroscience-based Nomenclature [75]. Given that dopaminergic gene sets, in our analysis, were associated with most of the investigated traits (Fig. 3), such a trans-diagnostic efficacy of dopamine-modulating drugs might be explained on the genetic level. It must be mentioned however that association of the DA gene set with a phenotype does not indicate any direction so that both enhancers, as well as inhibitors of dopamine neurotransmission, are effective in distinct phenotypes. Further genetic studies might refine such findings in that phenotypes characterized by hypo-, hyper-, and mixed dopaminergic states can be discerned, and these phenotypes might well cut across traditional phenotypes. Given the broad, unspecific efficacy of drug targeting the monoaminergic system, genetic studies might also help to identify drug target that shares efficacy but add specificity.

Some strengths and limitations of our study should be discussed. (i) We performed a systematic study of the genes encoding key proteins for the dopaminergic and serotonergic neurotransmitter systems in eight psychiatric disorders and the meta-analysis of all of them. At gene level, we identified twelve genes significantly associated with more than one disorder. However, none of them was associated with more than two disorders, being BIP and/or SCZ one of these conditions in most cases (10 out to 12). This brings up the issue that these two disorders could drive some of the results obtained because of their large sample sizes. (ii) Also, some individual genes could be involved in other neurotransmitter systems and several neuronal functions. Therefore, we decided to perform a combined analysis to study the contribution of genes enriched in DA or SERT pathways to the pathophysiology of these disorders using a competitive gene-set analysis. Moreover, DA and SERT neurotransmission are functionally interconnected, with several genes participating in both pathways, and this could have some impact on the results obtained in the gene-set analysis. In addition, it is important to note that we might be missing information from some genes as these pathways are still not very well defined, and also because the X-chromosome genes are not included in most GWAS. (iii) As we know, assigning SNPs to genes based on their location with respect to those genes is not the best approach, and for that reason we also performed a more functional analysis on Metaxcan. However, as this software only provides information about those gene models where the predictive power is good enough, we could not retrieve information from most DA and SERT genes, especially in brain tissues. (iv) Finally, no significant results were found for ANO, OCD, and TS, neither in the gene-based nor in the gene-set analyses, maybe due to limited sample sizes. In addition, this study was performed on individuals with European ancestry, so, further studies should be performed in larger samples and in other populations to confirm these results.

To our knowledge, this is the first systematic genetic study of the dopaminergic and serotonergic neurotransmitter systems in eight psychiatric disorders (ADHD, ANO, ASD, BIP, MD, OCD, SCZ, and TS) and in the meta-analysis of all of them. At gene level, we identified association of 67 DA and/or SERT genes with at least one of the studied disorders, twelve of them associated with two conditions. Gene-set analysis revealed significant associations with the DA gene sets for ADHD, ASD, and the cross-disorder GWAS meta-analysis, and with SERT gene sets for MD and BIP. The results obtained support a cross-disorder contribution of these two neurotransmitters systems in several psychiatric conditions.

## Supplementary information

Supplementary tables S1 to S18
